# Editorial: Applications of lung ultrasound in neonatology and pediatrics

**DOI:** 10.3389/fped.2024.1370969

**Published:** 2024-02-01

**Authors:** Stefano Nobile, Francesco Raimondi

**Affiliations:** ^1^ Neonatal Intensive Care Unit, Department of Mother, Child and Public Health, Fondazione Policlinico Universitario Agostino Gemelli IRCCS, Rome, Italy; ^2^ Neonatal Intensive Care Unit, Department of Translational Medical Sciences, Università “Federico II” di Napoli, Naples, Italy

**Keywords:** neonates, children, thoracic ultrasound, diaphragm, patent ductus arteriosus, lung aeration, feeding competence, bronchiolitis

**Editorial on the Research Topic** Applications of lung ultrasound in neonatology and pediatrics

Lung ultrasound (LUS) is a frequently used diagnostic tool in everyday clinical practice in Neonatology and Pediatrics, in contrast to the previous idea that lungs cannot be studied by ultrasound as air is not able to reflect ultrasound. However, the pioneering research by Lichtenstein and colleagues (1) revealed the significance of reverberation artefacts behind the pleural line and other ultrasound images. Since then, LUS has been increasingly used thanks to its advantages, which largely overcome its potential disadvantages (e.g., local availability of ultrasound machines and probes, costs, need for training, interference with minimal handling practices in preterm infants). Another benefit of LUS implementation in clinical practice is a significant reduction of radiation exposure, which is particularly significant for this vulnerable population (2). Considering the long life expectancy of infants, perinatal exposures (including radiation exposure) and conditions may influence the development of later (pediatric and adult) diseases, as proposed by Developmental Origins of Health and Disease hypothesis (3).

In particular, LUS has good diagnostic accuracy for several neonatal diseases and is useful to guide treatment (i.e., surfactant for respiratory distress syndrome, pleural drainage for pneumothorax and pleural effusions), to enable a faster diagnosis of potentially critical diseases (i.e., pneumothorax), to predict bronchopulmonary dysplasia and monitor the evolution of some conditions over time (i.e., chronic lung disease, pneumonia).

One of the most important applications of LUS includes the assessment of respiratory distress syndrome (RDS), a frequent neonatal condition currently treated with endotracheal surfactant administration based on the degree of oxygen need in the first hours of life (the optimal threshold being still unclear) (4, 5). LUS has been shown to be a useful tool to monitor the respiratory status during RDS as the LUS trajectory is gestational age dependent, significantly correlates with the oxygenation status, and predicts the onset of bronchopulmonary dysplasia (6).

In recent years, the utility of LUS has been established for other neonatal and paediatric conditions: transient tachypnoea of the newborn, pneumothorax, bronchopulmonary dysplasia, pneumonia, pleural effusion (7–10).

In this Research topic, recent and novel applications of LUS were reported. Dassios et al. sought to investigate for the first time the relationship between patent ductus arteriosus (PDA) and diaphragmatic dysfunction, two conditions which are frequently observed in neonates (11). The authors used point of care ultrasound to compare diaphragmatic function in infants with a PDA compared to those without a PDA. The M-mode ultrasonography was used to measure the mean inspiratory velocity, an index of inspiratory muscle fatigue: this parameter was significantly lower in the scans with a PDA compared to those without a PDA after adjusting for differences in gestational age and other confounders, suggesting that the presence of a PDA negatively influences diaphragmatic contractility. The authors proposed that diaphragmatic dysfunction with concomitant PDA may partially explain the failure of extubation success in preterm infants.

In another paper, Pryor et al. provided data on lung aeration after birth in an experimental model of RDS. The authors used LUS to describe the trajectory of lung aeration and airway liquid clearance in lambs with a low (controls) or high (elevated lung liquid levels) risk of developing RDS. They found a good correlation between LUS and gas exchange, and showed that that the trajectory of lung liquid clearance is slower in lambs with elevated liquid levels at birth. In addition, the authors found that the qualitative and quantitative LUS analysis assessed by pixel intensity variation could detect differences in lung aeration that cannot be readily identified by using qualitative LUS grading systems.

Controzzi et al. explored a novel concept, namely the potential role of LUS for the development of feeding competence in preterm infants. Since these infants are at increased risk of silent microaspirations, they received serial LUS scan before and after feeds starting from the first days of life until discharge. Contrary to what was expected, the authors reported no differences in LUS scores before and after feeds, and that the achievement of oral feeding competence was associated with gestational age at birth but not to LUS scores. The authors showed that the introduction of the first meal by bottle was associated either with gestational age and LUS score, and confirmed the role of LUS in predicting the duration of respiratory support and oxygen supply.

Finally, De Rose et al. compared for the first time the LUS features from neonates and infants up to 3 months of age with bronchiolitis caused by a single viral infection vs. coinfections. In contrast to their hypothesis, the authors reported that infants with a single viral infection and those with coinfections had similar LUS scores. LUS was nonetheless useful to assess prognosis, as an LUS score higher than 8 significantly predicted the need for any respiratory support, whereas an LUS score higher than 13 significantly predicted the need for mechanical ventilation.

In conclusion, LUS is increasingly being used for diagnostic and therapeutic applications in Neonatology and Pediatrics. Advantages of LUS include its availability, ease of use, favorable learning curve, reduction of radiation exposure. Recent research has widened the horizon of LUS applications, as provided in this Research Topic.
